# Treatment Progress of Immune Checkpoint Blockade Therapy for Glioblastoma

**DOI:** 10.3389/fimmu.2020.592612

**Published:** 2020-11-30

**Authors:** Na Zhang, Li Wei, Meng Ye, Chunsheng Kang, Hua You

**Affiliations:** ^1^ Medical Oncology Department, Affiliated Cancer Hospital and Institute of Guangzhou Medical University, Guangzhou, China; ^2^ Medical Oncology Department, The Affiliated Hospital of Medical School of Ningbo University, Ningbo, China; ^3^ Laboartory of Neuro-oncology, Tianjin Neurological Institute, Key Laboratory of Post-Neuroinjury Neuro-repair and Regeneration in Central Nervous System, Department of Neurosurgery, Tianjin Medical University General Hospital, Tianjin, China

**Keywords:** glioblastoma, immunotherapy, checkpoint inhibitors, checkpoint blockade therapy, resistance mechanism

## Abstract

Glioblastoma (GBM) is a highly malignant and aggressive primary brain tumor mostly prevalent in adults and is associated with a very poor prognosis. Moreover, only a few effective treatment regimens are available due to their rapid invasion of the brain parenchyma and resistance to conventional therapy. However, the fast development of cancer immunotherapy and the remarkable survival benefit from immunotherapy in several extracranial tumor types have recently paved the way for numerous interventional studies involving GBM patients. The recent success of checkpoint blockade therapy, targeting immunoinhibitory proteins such as programmed cell death protein-1 and/or cytotoxic T lymphocyte-associated antigen-4, has initiated a paradigm shift in clinical and preclinical investigations, and the use of immunotherapy for solid tumors, which would be a potential breakthrough in the field of drug therapy for the GBM treatment. However clinical trial showed limited benefits for GBM patients. The main reason is drug resistance. This review summarizes the clinical research progress of immune checkpoint molecules and inhibitors, introduces the current research status of immune checkpoint inhibitors in the field of GBM, analyzes the molecular resistance mechanism of checkpoint blockade therapy, proposes corresponding re-sensitive strategies, and describes a reference for the design and development of subsequent clinical studies on immunotherapy for GBM.

## Introduction

Glioblastoma (GBM) is the most advanced WHO grade IV glioma and the most common adult astrocytoma. GBM patients generally have a median survival of less than 20 months, and the 5-year survival rate is only 4–5% (1).The survival of GBM patients has not improved significantly over the past three decades. Despite aggressive standard treatments of maximum safe surgical resection, radiotherapy, and temozolomide in patients, the prognosis in newly diagnosed patients with GBM remains poor (2). GBM treatment, one of the most expensive therapy with least rewarding, is imposing a huge burden on the society. Hence, the need for a more effective antitumor treatment has become the goal of researchers worldwide. In recent years, immune checkpoint inhibitors have been widely used as a crucial therapy for malignant tumors such as melanoma and lung cancer, leading to the provision of new research directions for the GBM treatment (3). The suppression of autoreactive T cells by immune checkpoints is a defensive measure against autoimmunity under physiological conditions. In pathological conditions, immune checkpoints protect tumor cells from immune system clearance in a similar way. Compared to the cytotoxic effects of traditional chemotherapeutics and traditional targeted therapy, immune checkpoint targeted therapy aims to regulate checkpoint molecules, change their functions, and induce the death of tumor cells (4).

The widespread application of immune checkpoint inhibitors in the field of oncology, brought new hope to humans. However, available data indicate that it is beneficial for some patients, whereas some patients progressed or relapsed after effective treatment for some time. Furthermore, some patients did not respond to immune checkpoint inhibitor treatment in the beginning, so drug resistance is the main reason for the failure of immune checkpoint blockade therapy (5). The resistance of immune checkpoint inhibitors can be divided into primary, adaptive and acquired resistance based on resistance time. Primary resistance means that the tumor does not respond to immune checkpoint inhibitor treatment in the beginning. Acquired resistance implies that the tumor is effective to immune checkpoint inhibitor treatment in the beginning, but the disease progresses or recurs after a period of treatment. Adaptive resistance means that the tumor can be recognized by the immune system, but the tumor cells adapt to the immune system without being attacked by the immune system (6). It can further be divided into endogenous resistance and exogenous. Endogenous resistance is caused by changes in tumor cells, such as alterations in immune recognition process, alterations in cell signaling pathways, alterations in gene expression, and DNA damage repair reaction. Exogenous resistance refers to external factors that might affect all the processes of T-cell activation.

This review summarized the mechanism of immune-checkpoint inhibitors, the characteristics of the GBM immune microenvironment, and the clinical research progress of immune checkpoint inhibitors in the GBM treatment. The molecular resistance mechanism of checkpoint blockade therapy is also discussed, and the corresponding re-sensitive strategies are proposed.

## Function of Immune Checkpoints

**Figure 1:**
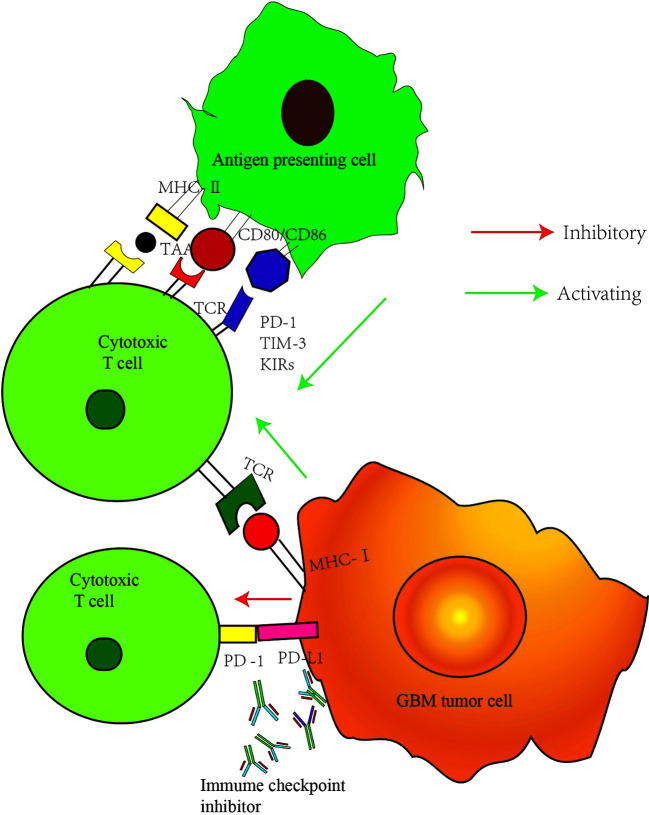
Major checkpoint inhibition pathway in GBM cancer cells. MHC II, major histocompatibility complex II; TAA, tumor associated antigen; TCR, T cell receptor; MHCI, major histocompatibility complex I; PD-1, programmed cell death protein 1; PD-L1, programmed cell death ligand protein 1; TIM-3, T cell immunoglobulin mucin molecule 3; KIRs, killer immunoglobulin-like receptors.

Immunotherapy is a therapeutic method that removes cancerous cells by improving the body’s autoimmune function. T cells play a vital role in antitumor immunity. The production of effector T cells and their recognition and elimination of cancerous cells are complex multi-step processes regulated by a series of activation and inhibition signals (7). The main function of inhibition signals is to prevent the overactivation of the immune system and the occurrence of uncontrolled inflammatory response and the autoimmune disease caused by it. Suppressing T-cell antitumor immune response, however, leads to the escape of cancer cells (8). Therefore, the elimination of cancer cells depends on the balance between the activation signal and the inhibition signals. Immune checkpoint receptors, such as cytotoxic T lymphocyte-associated antigen-4 (CTLA-4) and programmed cell death protein-1 (PD-1) expressed on the T-cell surface, play a negative regulatory role during the process of T-cell activation, thereby preventing pathological over activation (9). Interfering with the immune checkpoint signals can improve the antitumor immune response by restoring T-cell function. CTLA-4 mainly acts at the early stage of immune activation, regulating the initiation and activation of T cells, and the anti-CTLA-4 antibody can activate T cells in peripheral lymphoid tissue. PD-1 mainly plays a role in the effect phase of the immune response (10). Its overexpression is observed during the activation of T cells stimulated by antigen (**Figure 1**). The interaction of PD-1 with its ligand (programmed cell death ligand protein-1) PD-L1 or (programmed cell death ligand protein-2) PD-L2 can inhibit the transduction of T-cell signals and cytokine production and reduce the number of T cells (9). These two ligands play an important role in the tumor microenvironment and are expressed in many cancer cells. Antibodies against PD-1 or PD-L1 can inhibit the transmission of this negative signal and restore cell function.

## Clinical Research Progress of Immune Checkpoint Inhibitors in GBM Treatment

Immune regulation depends on the balance between the activation and inhibition signals. In the physiological state, immune checkpoint molecules can inhibit cytotoxic T-cell function as an immunomodulatory mechanism (11). When the immune checkpoint is abnormal or continuously activated, the tumor immune response is suppressed, and the monoclonal antibody against the immune checkpoint can release the “immune brake,” leading to the enhancement of the immunotherapy effect (12). Currently, related checkpoints are mainly focused on PD1 and CTLA-4. Although significant results (e.g., melanoma) have been obtained in clinical trials involving solid tumors, studies involving checkpoint inhibitors for GBM treatment are still being conducted.

Clinical trials on immune checkpoint inhibitors are mainly divided into the following categories: immune checkpoint inhibitor monotherapy and combination therapy. Combination therapy includes immune checkpoint inhibitors combined with chemotherapy, stereotactic radiosurgery therapy, or targeting other immune targets. Clinical trials of GBM immune checkpoint inhibitors are still in the early stage. Most trials are in the recruitment stage or in progress (**Table 1**), with only a few published preliminary results. Currently, there are seven immune checkpoint inhibitors approved for sale in the United States, including one monoclonal antibody against CTLA-4 (Ipilumumab), three monoclonal antibodies against PD-1 (Nivolumab, pembrolizumab, and Cemiplimab), and three PD-L1 monoclonal antibodies (atezolizumab, Devaru, and Avelumab). Some of them have already been measured in some clinical trials. Schalper et al. treated 30 patients with GBM (3 cases of primary GBM and 27 cases of recurrent GBM) with Nivolumab (trial no. NCT02550249) before and after operation (13). Adjuvant Nivolumab therapy can enhance chemokine transcription, increase immune cells infiltration, and increase T-cell receptor Crohn-like in the tumor microenvironment. However, there was no significant survival benefit in 27 patients with recurrent GBM, but 2 of 3 patients with primary GBM survived for 28 and 33 months, respectively. Another phase III clinical trial of Ipilumumab combined with Nivolumab (trial no. NCT02017717) is also under way, Forty recurrent GBM patients were randomly divided into two groups: Nivolumab group and Nivolumab + Ipilimumab group. The results showed that the tolerance of patients in Nivolumab group was good, whereas the tolerance of patients in Nivolumab + Ipilimumab group was affected by an excessive dose of Ipilimumab (14). The subsequent phase III trial of CheckMate 143 showed that Nivolumab did not show more significant survival benefits than bevacizumab (median overall survival time 9.8 vs. 10 months) (15). The trail’s failure may be related to the low expression level of PD-L1 in the included patients (16). Although few clinical trials on GBM immune checkpoint inhibitors have been successful, researchers have never given up. New potential immune checkpoints such as dioxygenase, CD47, and CD137 have been found, providing the possibility of successful immunotherapy in the future. A recently published phase I single-arm clinical trial (trial no. NCT02658981) included 44 GBM patients treated with lymphocyte activation gene (LAG) inhibitor, CD137 inhibitor, and the combination with a checkpoint inhibitor, to explore treatment strategies for potential targets. The results have not yet been published yet.

**Table 1:** Currently ongoing clinical trials based on immune checkpoint inhibitors*.

Clinical Trial	Phase	Study population	Target	Experimental design
NCT02017717 (Check Mate-143)	III	Recurrent GBM	PD-1 VEGF	Nivolumab vs. bevacizumab (phase III), nivolumab vs. ipilimumab + nivolumab (phase I)
NCT02617589 (Check Mate-498)	III	Primary diagnosed GBM *MGMT*-unmethylated	PD-1	Nivolumab + radiotherapy VS. TMZ+ radiotherapy
NCT02667587 (Check Mate-548)	III	Primary diagnosed GBM *MGMT*-unmethylated	PD-1	Nivolumab + TMZ+ radiotherapy VS TMZ+ radiotherapy
NCT03726515	I	Newly diagnosed GBM *MGMT*-unmethylated	PD-1	CAR-EGFRvIII-T cell + Pembrolizumab
NCT02550249	III	Primary GBM Recurrent BGM	PD-1	Nivolumab group vs. Nivolumab + Ipilimumab group
NCT03707457	I	Recurrent BGM	PD-1 IDO1	Nivolumab Anti-GITRantibody MK-4166 IDO1inhibitory INCB024360 Ipilimumab
NCT02852655	II	Recurrent GBM	PD-1	Neoadjuvant and postsurgical pembrolizumab vs. postsurgical pembrolizumab alone
NCT03743662	II	Recurrent GBM *MGMT*-methylated	PD-1 VEGF	Nivolumab BEV
NCT02658981	I	Recurrent GBM	PD-1 LAG-3 CD137	Nivolumab BMS986016(anti-LAG-3antibody) Urelumab(anti-CD137antibody)
NCT03233152	I	Recurrent GBM	PD-1 CTLA-4	Nivolumab + Ipilimumab
NCT02287428	I	Primary diagnosed GBM *MGMT*-unmethylated	PD-1	Pembrolizumab + Personalized neoantigen vaccine (NeoVax) vs. radiotherapy +NeoVax
NCT02335918	II	Recurrent GBM	PD-1 CD27	Anti-CD27antibody Varlilumab + Nivolumab
NCT03493932	I	Recurrent GBM	PD1 LAG-3	Nivolumab BMS986016
NCT02968940	II	Recurrent IDH mutant GBM	PD1	Avelumab
NCT03422094	I	Primary diagnosed GBM *MGMT*-unmethylated	PD-1 CTLA-4	NeoVax Nivolumab Ipilimumab
NCT03491683	I/II	Primary diagnosed GBM	PD-1	IN0-5401+ IN0-9012 + Nivolumab + Cemiplimab + TMZ
NCT03718767	II	Recurrent IDH mutant GBM	PD-1	Nivolumab
NCT02798406	II	Recurrent GBM	PD-1	Oncolytic virus DNX-2401 Pembrolizumab
NCT03341806	I	Recurrent GBM	PD-L1	Avelumab
NCT03291314	I	Recurrent GBM	PD-L1 VEGFR	Avelumab + Axitinib
NCT02794883	II	Recurrent GBM	PD-L1 CTLA-4	Durvalumab Anti-CTLA-4 antibody Tremelimumab
NCT02336165	II	GBM	PD-L1 VEGF	Durvalumab + radiotherapy (newly diagnosed GBM), durvalumab monotherapy (recurrent GBM), durvalumab + bevacizumab (recurrent GBM)
NCT03047473	II	Primary diagnosed GBM	PD-L1	Avelumab +TMZ
NCT02311920	I	Primary diagnosed GBM	PD-1 CTLA-4	Nivolumab Ipilimumab TMZ
NCT04003649	I	Recurrent BGM	PD-1 CTLA-4	CAR-T cell + Nivolumab + Ipilimumab vs. CAR-T cell + Nivolumab
NCT04047706	I	Primarydiagnosed GBM	PD-1 IDO1	IDO1inhibitory BMS986205+Nivolumab+ TMZ + radiotherapy vs. IDO1inhibitory BMS986205+ Nivolumab + radiotherapy

GBM, glioblastoma; PD1, programmed cell death protein 1; VEGF, vascular endothelial growth factor; BEV, bevacizumab; TMZ, temozolomide; EGFRvⅢ, epidermal growth factor receptor variant Ⅲ; IDO1, indoleamine-2, 3-dioxygenase 1; GITR, glucocorticoid-induced tumor necrosis factor receptor; LAG-3, lymphocyte-activation gene 3; DC, dendritic cells; CTLA-4, cytotoxic T lymphocyte-associated antigen 4; PDL1, programmed cell death protein ligand 1; IDH, isocitrate debydrogenase; CAR, chimeric antigen receptor.

*All the data come from ClinicalTrials.gov.

## Analysis of Resistance Mechanism in Immune Checkpoint Inhibitor Treatment

**Figure 2:**
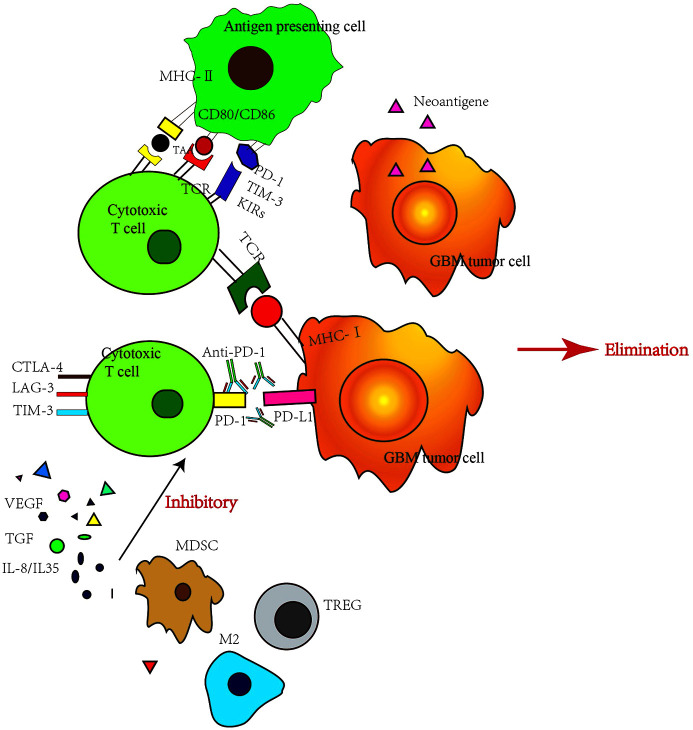
Immune checkpoint inhibitor resistance mechanisms. VEGF, vascular endothelial growth factor; TGF, transforming growth factor; MDSC, myeloid-derived suppressor cell; M2, M2 type macrophage; TREG, regulatory T cell; LAG-3, Lymphocyte activation gene-3.

T-cells’ activity can be inhibited by some small molecular proteins. Tumor cells use this mechanism to suppress T-cells and survive by escaping from the human immune system. Immune checkpoint inhibitors can relieve this inhibition, reactivate T-cells and destroy cancer cells. Thus, T-cells play a vital role in this process, not only T-cells themselves, but also factors secreted by multiple originated tumor associate microenvironment (TAM) (17). TAM consist by tumor-associated myeloid cells, cancer stem cells, fibroblasts, other permeable immune cells and cells which form vessels or lymphatics. The immune suppressive microenvironment of GBM patients is a comprehensive and self-sufficient system (18). The drug resistance of immune checkpoint inhibitor is complex program, which can be divided into endogenous resistance and exogenous resistance. Endogenous resistance refers to drug resistance caused by changes in tumor cells, such as changes in the immune recognition process, cell signaling pathway, gene expression, and DNA damage repair. Exogenous resistance means that all processes of T-cell activation are affected by external factors (19). The mechanisms and strategies of overcoming various resistance are described below (**Figure 2**).

Intrinsic factors refer to tumor cells expressing certain genes or inhibiting certain signal transduction pathways, preventing immune killer cells from infiltrating or playing a role in the tumor microenvironment, leading to immunotherapy resistance (20). The recognition of tumor antigens by effector T cells is particularly important in immunotherapy. When the mutation load of the tumor and the ratio of DNA mismatch repair and genomic microsatellite instability-high(MSI-H) are low, the production of tumor-associated antigens is reduced, which may cause drug resistance (21). Generally, low mutation burden is one reason why GBM is insensitive to immunotherapy. However, high hypermutation is observed in some gliomas cases, and chemotherapy can drive the acquisition of hypermutated populations without promoting a response to PD-1 blockade. In lung cancer and melanoma, the high tumor mutation burden(TMB) was originated from the accumulation of clonal mutations during the longstanding process of tumor generating. However, the use of TMZ in newly diagnosed gliomas with low TMB resulted in the selection pressure, further induced the resistant clones generation (MMR-deficient clones) with high TMB in a short period. Although these TMZ resistant gliomas cells obtained hypermutation, few clonal antigens per cell developed, thus no stronger immunogenic response was induced. So MMR-deficient hypermutation gliomas are characterized by a lack of prominent T cell infiltrates, extensive intratumoral heterogeneity, poor patient survival, and a low rate of response to PD-1 blockade (22).

The activation of the signaling pathway of mitogen-activated protein kinase, and the vascular endothelial growth factor and interleukin (IL) producing can inhibit the recruitment and function of T cells leading to the prevention of T-cell infiltration in the tumor (23). The deletion of the PTEN gene can increase the expression of immunosuppressive cytokines, leading to the reduction of T cell infiltration in tumors, and reducing T-cell-mediated tumor cell death. Thus, the PTEN gene deletion may promote immune tolerance (24). The interferon-γ (INF-γ) pathway plays a role in primary (25), adaptive and acquired resistance. INF-γ produced by tumor-specific T cells can recognize tumor cells and their homologous antigens and promote the increased expression of some protein molecules such as major histocompatibility complex (MHC) molecules and molecules involved in antigen presentation, molecules recruiting immune cells, and the effector molecules that inhibit tumor proliferation or promote tumor apoptosis. Therefore, tumor cells lacking the INF-γ signaling pathway are not vulnerable to T cells, leading to immune checkpoint inhibitor resistance (26).

The most important external factor is the immune microenvironment (27). Many immune cells are often gathered inside and around tumor cells, and these immune cells form a protective barrier against tumor (28). However, once this barrier is broken, there is an acceleration in the tumor occurrence and development. For example, regulatory T-cell (Tregs) play a major role in maintaining self-tolerance (29). Tregs can secrete inhibitory cytokines such as IL-10, IL-35 and transforming growth factor-β (TGF- β), or directly inhibit Teff (CD4+CD25-effector T-cells) response. Tregs can infiltrate various tumor cells (30). An experimental study has shown that the therapeutic effect of a CTLA-4 inhibitor is related to the ratio of Teffs to Tregs (31). The higher the ratio, the better is the therapeutic effect. Myelogenic inhibitory cells (myeloid-derived suppressor cells, MDSCs) represent a group of heterogeneous myeloid cells (32), which can strongly inhibit the antitumor activity of T cells, natural killer cells and some bone marrow cells such as dendritic cells, and stimulate the increase of Tregs. MDSCs also effect on neovascularization, tumor cell infiltration and metastasis, leading to tumor progression (33).

To improve the efficacy of immune checkpoint inhibitors in clinical treatment, there is an urgent need to find biomarkers that can predict treatment sensitivity and screen the population suitable for this therapeutic procedure. The key to the efficacy of immune checkpoint inhibitors lies in the effector immune cells reaching the tumor area. The immune checkpoint pathway plays a leading role in the mechanism of inhibiting anti-tumor immunity (34). The former is often judged by the expression of tumor-infiltrating lymphocytes (TILs) or the ratio of immune effector cells to immunosuppressive cells. However, the criteria for the latter are not clear enough, as no accurate biomarkers have been found. Currently, the most promising approach is the prediction of sensitivity toanti-PD-1/PD-L1 therapy (35). Clinical studies on melanoma have shown that the density of TILs and the proportion of T cells expressing PD-1 or PD-L1 are related to the sensitivity to treatment. According to these indicators, tumors are divided into four types. Type I tumors (TILs+, PD-L1+) which exhibit adaptive immune resistance, are most likely to be sensitive to immune checkpoint inhibitors. Type II tumor (TILs-,PD-L1-) are characterized by immunological ignorance and likely to be insensitive to immune checkpoint inhibitors due to the absence of an obvious immune response. Type III tumors (TILs-,PD-L1+) show intrinsic induction, which is the tumor intrinsic expression of PD-L1 in the absence of immune response. This type of tumor is ineffective when immune checkpoint inhibitors are used alone. It also emphasizes that PD- L1expression cannot be used alone as an index to predict the efficacy of PD-1/PD-L1 inhibitors. Type IV tumors (TILs +, PD-L1-) are characterized by immune infiltration tolerance. It does not depend onPD-L1 expression. Other immunosuppressive signals may exist in this type of tumor, so that the inhibition of other immune checkpoints may have a therapeutic effect. Although this classification is based on the study of melanoma, it provides a theoretical basis for understanding the tumor immune microenvironment of GBM and the rational use of immune checkpoint inhibitors. However, the efficiency and reliability of predicting the sensitivity of GBM to immune checkpoint inhibitors need further research. In addition, the combination of immune checkpoint inhibitors and other antineoplastic drugs is also under study (36). Chemotherapy, radiotherapy, kinase inhibitors, and epigenetic modified drugs may have a synergistic effect on immunotherapy by improving tumor immunogenicity (27).

## Conclusions

There is no U.S. Food and Drug Administration-approved immunotherapy for GBM despite numerous unique therapies currently tested in clinical trials. GBM is a highly immunosuppressive tumor and there are limitations to the extent of a safe immune response in the central nervous system. To date, many trials of targeted therapies comprising single components have not demonstrated any significant efficacy in GBM treatment. The advent of immune checkpoint inhibitors has led to the improved prognosis of many patients with solid tumors, such as malignant melanoma, non-small cell lung cancer, and renal cell carcinoma. However, it has only limited efficacy in clinical trials of GBM. To improve the efficacy of immune checkpoint inhibitors in treatment GBM, there is a need for biomarkers that can effectively predict the effect of immunotherapy to screen the adaptive patients to achieve “individualized immunotherapy” (37). Immune checkpoint inhibitors may have a lasting clinical effect in a small number of patients. To reduce or delay drug resistance, the combination of multiple treatment strategies is encouraged. The main therapeutic markers currently used include PD-L1 expression, tumor mutation burden, TILs, and MSI-H. However, due to the complexity of the antitumor immune response and the huge heterogeneity of tumors, the prediction of a curative effect and the screening of markers are still difficult and challenging. Whole-genome sequencing and epigenetic analysis help select the dominant population and perform an accurate, individualized treatment. Conversely, the combination of other anticancer therapies is also expected to produce a synergistic effect. In combination with tumor gene analysis and immune characteristic analysis, making full use of the synergistic effect of different treatment strategies to carry out combination therapy is a feasible measure in reducing or delaying immune resistance in checkpoint inhibitor drugs.

## Author Contributions

NZ, LW, MY, CK, and HY conceived the hypothesis, did the literature search, and wrote the manuscript. All authors contributed to the article and approved the submitted version.

## Funding

The research work in the lab is supported by the National Natural Science Foundation of China (No. 81911530169, 81903088, 81850410547, and 81670180). The funders had no role in study design, data collection and analysis, decision to publish, or preparation of the manuscript.

## Conflict of Interest

The authors declare that the research was conducted in the absence of any commercial or financial relationships that could be construed as a potential conflict of interest.
